# Opportunities for the future of transcranial magnetic stimulation and epilepsy

**DOI:** 10.1016/j.neurot.2026.e00947

**Published:** 2026-06-15

**Authors:** Brian N. Lundstrom, Alexander Rotenberg

**Affiliations:** a Department of Neurology, Mayo Clinic, Rochester, MN, USA; b Department of Neurology, Boston Children's Hospital, Boston, MA, USA

**Keywords:** Epilepsy, TMS, Non-invasive brain stimulation, Neuromodulation

## Abstract

Drug-resistant epilepsy has traditionally been managed with medications or surgery aimed at removing or destroying epileptogenic tissue. In contrast, electrical brain stimulation seeks to alter dysfunctional neural circuits without tissue destruction. Transcranial magnetic stimulation (TMS) is a particularly attractive approach due to its favorable side-effect profile and robust diagnostic as well as therapeutic capacity. Cortical stimulation by TMS generates quantifiable evoked potentials that provide insights into seizure susceptibility and target engagement by therapeutics. Repetitive TMS (rTMS) has promise as a method for treating seizures, but essential questions remain as to which epilepsy syndromes and which cortical targets will respond to stimulation. Here, we speculate on questions pertaining to diagnostic and therapeutic TMS that may be answered in near-future studies. Will reliable measures to quantify seizure likelihood be available from measures of TMS-EMG or TMS-EEG evoked potentials? Might TMS help optimize other neuromodulation techniques? Can rTMS targeting superficial cortex, either in isolation or coupled with appropriately selected drugs, suppress generalized onset or multifocal seizures? Recent advances highlight the potential of bioelectronic medicine approaches such as TMS.

## Non-invasive brain stimulation in epilepsy care and research

Non-pharmacologic approaches for treating drug-resistant epilepsy typically focus on epilepsy surgery and related diagnostics aimed at removing or destroying brain tissue. To complement destructive therapies, electrical modulation of brain activity is now often considered [[1], [2], [3]]. Broadly, neuromodulation can be divided into invasive and non-invasive protocols. Non-Invasive Brain Stimulation (NIBS) in particular holds special appeal [4] given its favorable safety profile, and potential in epilepsy diagnostics as well as epilepsy therapeutics. In addition, NIBS has the advantage of extremely low morbidity or risk, and has been explored extensively as a therapeutic and diagnostic tool in range of neuropsychiatric diseases beyond epilepsy [5,6].

**Fig. 1:**
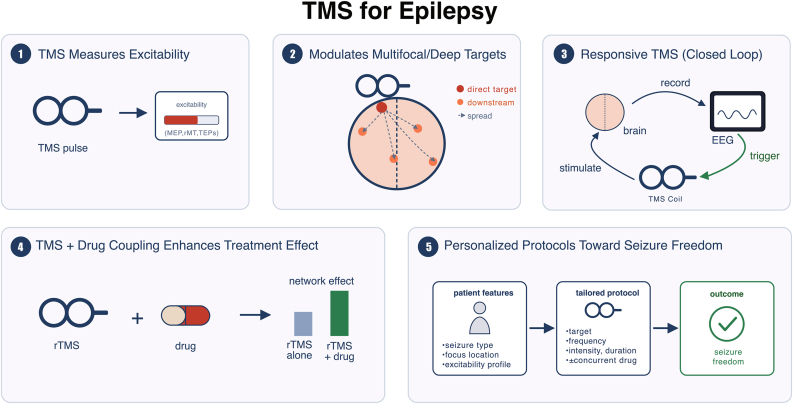
Opportunities for TMS in the diagnosis and treatment of epilepsy.

NIBS incorporates a variety of technologies ranging from the inexpensive transcranial direct current stimulation (tDCS) devices sold directly to consumers that deliver very weak, long, broad currents through scalp electrodes [7,8] to more sophisticated and expensive transcranial magnetic stimulation (TMS) devices that deliver strong, brief pulses of electrical current to focal brain regions and elicit evoked responses [9]. Emerging NIBS technologies also include focused ultrasound (FUS) systems that utilize neuro-navigation guided targeting to non-invasively modulate specific brain regions with spatial precision approaching that of invasive approaches, but without requiring surgical implantation [10]. Of these techniques, the most often cited related to epilepsy is TMS. Here, we discuss plausible TMS roles in epilepsy, including advanced assessments of cortical excitability, treating multifocal or generalized epilepsy, and the potential for seizure freedom (Fig. 1). We speculate on near future opportunities for TMS related to epilepsy networks and seizure management.

## Supposition 1: TMS evoked responses will increasingly be used as markers of cortical excitability

An especially practical diagnostic application of TMS is the use of evoked responses to assess cortical excitability. When delivered to the primary motor area and coupled with electromyography (EMG), TMS can generate motor evoked potentials (MEPs) that can be recorded with surface electrodes over a contralateral limb. When at rest, the MEP amplitude at a fixed TMS strength, or the TMS strength required to generate an MEP of a predetermined size, defines the resting motor threshold (rMT), which provides a convenient output measure to assess brain excitability [11,12]. Since the primary motor neocortex shares many similarities with other neocortical regions, and is extensively connected to numerous brain areas, the rMT provides a standardized metric of cortical excitability.

The rMT is increased by some classes of anti-seizure medications, and thus provides a measure of target engagement for developing therapeutics [13]. Voltage-gated sodium channel blockers, for instance, predictably increase the rMT [14,15]. In a recent example, ingestion of Xen1101 (a Kv7.2/Kv7.3 positive allosteric modulator) increases the rMT in direct correlation with its serum concentration. Similarly, using TMS to determine rMT can help distinguish responders from non-responders to anti-seizure medication treatment [16,17], which indicates prospects for TMS measures in drug dose or class selection in disease populations.

rMT can also provide insights into epilepsy physiology. In generalized epilepsy, the rMT is reduced bilaterally while it is reduced ipsilateral to the seizure focus for focal epilepsy [18], consistent with lateralization of excitability. Overall, the rMT has been used as an accessible threshold for cortical recruitment that is often relevant brain-wide.

In addition to rMT, paired-pulse TMS (ppTMS) measures can probe the excitability of various time intervals following the initial stimulation pulse, providing a systematic way of evaluating evoked response dynamics [9]. For example, ppTMS in patients with generalized epilepsy shows increased facilitation at interstimulus intervals of 200–300 ms [18], which corresponds to the mean inter-discharge interval of spike-wave discharges on EEG, suggesting a lack of inhibition. This suggests that TMS can probe the temporal dynamics of pathological network oscillations. Since such oscillations may be affected by invasive stimulation techniques [19], TMS-derived excitability measures can provide a ready means to optimize invasive neuromodulation devices. Notably, while most often used to assess neural circuit properties in the motor cortex, ppTMS can be used with TMS-EEG to assess other cortical areas as well [20].

Generally, determining the optimal setting for invasive neurostimulation is challenging and driven by empirical approaches. Programming of invasive stimulation devices relies largely on seizure counts over weeks to months, an inherently slow and imprecise process. TMS could offer a rapid, objective readout of network-level effects of stimulation setting changes. For example, immediately after VNS was turned on, paired-pulse TMS found clear increases in inhibition (as measured by short-interval intracortical inhibition or SICI) but not excitation (as measured by rMT) [21]. This finding suggests that TMS can assess short-term GABAergic target engagement of VNS and suggests an approach by which VNS parameters could optimized. Deep brain stimulation (DBS) parameter adjustments could similarly be monitored by serial TMS-EMG, with the goal of identifying stimulation settings that optimally normalize cortical excitability. Longitudinal TMS studies have shown that seizure freedom is accompanied by normalization of TMS-derived cortical excitability metrics, while drug-resistant epilepsy is associated with progressively increasing excitability over years [22]. So, rather than waiting months to assess clinical seizure frequency, clinicians could use TMS-derived cortical excitability as an objective, near-real-time biomarker to guide optimization of stimulation parameters.

Another compelling prospective application of TMS excitability measures is in predicting which patients will develop epilepsy after traumatic brain injury (TBI) or stroke. Post-traumatic epilepsy (PTE) develops in 10–20% of patients with significant TBI, yet there are currently no validated biomarkers to identify at-risk individuals during the latent period between injury and seizure onset. Preclinical work has demonstrated that paired-pulse TMS can track the progressive loss of cortical inhibition that follows TBI [23,24]. In a fluid-percussion injury model, ppTMS revealed a progressive decline in intracortical inhibition beginning as early as two weeks post-injury, paralleling increasing oxidative stress, degradation of perineuronal nets, and loss of parvalbumin-positive inhibitory interneurons. These findings establish ppTMS as a translational biomarker capable of tracking the trajectory of cortical inhibitory dysfunction during epileptogenesis.

In post-stroke epilepsy, TMS studies have similarly revealed heightened cortical excitability in the affected hemisphere, i.e., larger MEP amplitudes and increased intracortical facilitation, in patients who developed seizures compared to stroke patients who did not [25]. These differences suggest that TMS may distinguish a “pro-epileptogenic” excitability profile after cerebrovascular injury. A clinical trial (NCT05517954) is underway to test the hypothesis that TMS-derived cortical excitability measures in TBI patients reflect heightened epileptogenic potential that can predict PTE development. Were such a biomarker validated, it could fundamentally alter clinical practice: serial TMS monitoring after TBI or stroke could identify a window of escalating excitability predictive of impending epileptogenesis, enabling targeted prophylactic intervention during the critical latent period before the first seizure occurs.

Although the MEP and rMT have been taken as a brain-wide or hemisphere-wide measure of cortical excitability, one need not limit TMS stimulation to the primary motor area. Other cortical areas can be targeted, although EEG is then needed to assess the results of the stimulation. A reasonable expectation for TMS-EEG protocols is that these may mimic cortico-cortical evoked potentials (CCEPs), or even thalamocortical evoked potentials [26], and can contribute to seizure onset zone localization or to other epilepsy diagnostics. Numerous reports describe TMS-evoked potentials (TEPs) [20,27] that can be acquired with TMS-EEG protocols, including a recently updated expert review [28]. TMS-EEG may be a non-invasive means to immediately evaluate treatment effects and changes to network connectivity [29] as well as distinguishing generalized epilepsy patients from normal subjects [30]. Yet, even with invasive EEG, the structure of evoked responses is unclear [31], and scalp-recorded TEPs may be yet more complex. While the focus has typically been on some measure of initial response amplitude, later TMS-EEG responses may be just as or more important [20,32]. Later responses are likely related to aspects of negative feedback mechanisms, which are emphasized in control theory approaches, and may play critical roles ensuring that the brain is neither too stable nor too excitable [33,34].

These complexities highlight some of the potential limitations of using TMS to assess cortical excitability: responses are a mix of cortical and subcortical responses, it is difficult to account for patient-specific anatomic differences and brain state changes, and cortical areas certainly to some extent differ. Despite the challenges of understanding complex TEPs and potential technical challenges related to accurately recording these responses [35], diagnostic TMS-related protocols could be of clinical benefit even today. A continuing challenge is that TEPs and epilepsy remain understudied, and many approaches need further development or validation. One example relates to aspects of ppTMS, where early evidence of utility has not been supported by later verification [36]. Nonetheless, our supposition is that TEP measures collected across a range of epileptic conditions and anti-seizure treatments will provide practical biomarkers of disease severity and target engagement measures.

## Supposition 2: Repetitive TMS can treat multifocal epilepsy or indirectly target deep regions of increased excitability

Therapeutic approaches for drug-resistant seizures have typically centered on focal epilepsy with a single focus, where destructive but curative procedures can be considered. Even with single focus epilepsy, additional considerations relate to how deep the focus is and how well it is localized. TMS coils often provide very focal stimulation to superficial cortical targets. Although some TMS coils are optimized for deeper foci (perhaps up to 6 cm), superficial areas are still stimulated and questions regarding the anti-seizure effectiveness of deep stimulation remain [[37], [38], [39], [40]]. Questions related to targeting become more complex when multifocal or generalized epilepsies are considered.

Even when targets are too deep or multifocal, might there be a proxy target or a set of targets that sufficiently modulate the epileptogenic network to suitably treat or even potentially stop seizures? Well-established data suggest that focal TMS treatment of the left dorsolateral prefrontal cortex (DLPFC) can improve symptoms of major depression [41], and yet the left DLPFC is not the primary, causative location for depression [42]. In epilepsy, there may be similar canonical stimulation target sites. Stimulation of the anterior nucleus of the thalamus (ANT) for seizures involving the limbic network is effective [43]. Perhaps the DLPFC could be an effective non-invasive stimulation target for limbic-associated seizures? In a cohort of patients with seizures characterized by generalized spike-wave discharges, the source of the EEG discharges was localized to the right frontal lobe [44]. Thus, perhaps right frontal stimulation could be considered as a target to suppress drug-resistant generalized onset seizures. Treating epilepsy network nodes is a prevalent theme of invasive stimulation that could be applied to NIBS and rTMS.

## Supposition 3: Responsive repetitive TMS will be more effective for seizure suppression

rTMS has typically been applied in a continuous or semi-continuous “open loop” manner. However, oscillations are a fundamental property of brain function. It is increasingly appreciated that the effect of external stimulation depends on these oscillations, and advanced methods for responsive, closed loop stimulation for TMS have been developed [45]. Emerging mechanistic insights from invasive closed-loop responsive neurostimulation suggest that therapeutic outcomes arise not from direct inhibition of seizures but rather from indirect network modulation over time [46]. In fact, stimulation during low-risk states predicts seizure reduction better than stimulation during high-risk states [47]. The cellular and molecular mechanisms underlying effects of NIBS are often related to plasticity-related mechanisms such as NMDA receptor-dependent long-term potentiation (LTP) and long-term depression (LTD) mechanisms [[48], [49], [50]]. The overlap between rTMS effects and LTP/LTD mechanisms is underscored by the capacity of NMDA receptor antagonists to abolish rTMS effects on motor evoked potential amplitude [48,51]. A logical extension of these observations is that rTMS modulatory effects are strongly dependent on underlying brain state, e.g., regional membrane polarization and NMDA receptor activation. Variable effects are often seen as rTMS-induced plasticity depends critically on pre-stimulation brain state, time of day, genetic factors, and individual network architecture [52]. Likely, brain stimulation gradually reshapes pathological network dynamics rather than suppressing seizures as they occur. Optimal stimulation timing may depend on brain state rather than seizure occurrence. Our supposition is thus that rTMS protocols where stimuli are gated by real-time EEG to assess brain oscillations and state will improve seizure suppression.

## Supposition 4: TMS-drug coupling will improve TMS effectiveness

Targeted use of medications during brain stimulation may enhance the anti-seizure NIBS effectiveness. In tDCS, for instance, deliberate targeting of NMDA-type glutamate receptor-mediated excitation appears synergistic for purposes of seizure control when combined with cathodal cortical stimulation [53] in preclinical models. rTMS also may work synergistically with lorazepam, as indicated in a rat seizure model [54]. Yet clinical testing of rTMS-drug combinations is absent. We anticipate that medications (e.g. d-cycloserine) that facilitate NMDA-type glutamate receptor-mediated plasticity may enhance brain stimulation effects [55,56]. In this sense, the purpose of medications would not be to chronically alter brain excitability but rather to transiently enhance plasticity thus potentiating rTMS anti-seizure effect.

One might imagine an “induction” drug-device protocol to promote appropriate cortical changes that will reduce seizure likelihood. A subsequent maintenance protocol might avoid medications and any related side effects and instead focus on more gradual changes, which perhaps may continue via at-home tDCS therapies [57,58] or similar low-risk easy-to-administer approaches.

## Supposition 5: Personalized TMS protocols will improve seizure freedom rates

Neuromodulation as a therapeutic approach for epilepsy is considered non-curative or palliative. However, there are clearly rare cases where seizure-freedom is obtained with ongoing stimulation and even rarer cases where it appears that a transient period of stimulation led to long-term seizure-free or near seizure-free states [59,60]. Is it possible that stimulation parameters (e.g. amplitude, frequency, target, duration, and even waveforms) could be optimized and personalized in ways that consistently lead to seizure-freedom?

Stimulation, at least at times, achieves maximal effectiveness over weeks or months, consistent with a mechanism of action that involves neuroplasticity. Could stimulation in part “teach” the brain to learn more helpful “anti-seizure” electrical patterns? Certainly, the brain is a learning machine, and even seizure-related activity appears at times to be solidified by mechanisms related to neuroplasticity [61]. If seizures can teach the brain to be more epileptic, then appropriately designed stimulation protocols, perhaps when combined with sleep optimization, medication-enhanced plasticity, and individualized targeting, might teach the brain anti-epileptic patterns.

We suggest that a reasonable goal of rTMS is seizure-freedom, that with proper markers of excitability to optimize stimulation parameters and approach, rTMS could be disease-modifying and effectively cure some patients. The demonstration of prolonged seizure-freedom following short-term TMS in individual cases, with persistent normalization of EEG biomarkers and spectral power months after stimulation cessation [60], provides proof-of-concept that transient NIBS can induce lasting network reorganization.

Related is the widely understood concept that seizures are only the tip of the iceberg as far as epilepsy morbidity. Seizures may appear paroxysmal, but seizure-related activity has ongoing effects on neural networks that are often detrimental. Increased anxiety, depressed mood, inability to focus, worsened memory, and poor sleep may all be intrinsically related to epilepsy. Certainly, anti-seizure medications are known to have wide-ranging effects on pain, anxiety, mood, memory, and sleep. DBS of the ANT can worsen mood while vagus nerve stimulation can improve mood; both typically improve seizures. Both TMS and tDCS are FDA-approved to treat depression [62,63] and could potentially affect these co-morbidities of epilepsy. For example, a recent meta-analysis suggests low-frequency rTMS not only reduces seizure frequency but also produces significant improvements in cognitive function in patients with epilepsy [64]. Especially given the favorable risk profile, could rTMS or tDCS be used to routinely simultaneously treat epilepsy and co-morbidities? Would treating depression improve seizure burden? Could multi-site treatment protocols be synergistic? The potential of NIBS is broad given the ready possibility that it could be used to treat a variety of targets, perhaps even changing targets, in a single person.

## TMS limitations

Notwithstanding potential uses in epilepsy, important limitations may hinder wider use of TMS. TMS is associated with a small risk of provoking or worsening seizures in patients with epilepsy [65]. Another concern is the paucity of reliability measures for TMS metrics leading to concerns of consistency over time. Both concerns can be addressed with targeted large prospective epilepsy studies. There are also physiologic limitations to TMS use, such as development-related changes. The rMT declines through childhood before stabilizing in adolescence and adulthood [16,66], which can translate to impractically high thresholds in very young children. This limitation may be addressed with developing technology that enables adjustable TMS pulse shape (particularly pulse width) that may be used to more efficiently activate the pediatric neocortex [67].

## Final thoughts

The landscape of TMS, and NIBS more generally, continues to evolve with novel technologies addressing limitations of current approaches. The integration of multimodal approaches, combining TMS with advanced EEG analysis, invasive recordings, computational modeling, and precision pharmacology, offers a path toward truly personalized epilepsy treatment. Machine learning analysis of individual patient network architecture, genetic factors affecting plasticity, circadian rhythms, medication responsiveness, and cognitive phenotypes could optimize stimulation parameters, timing, targets, and adjunctive therapies to maximize effectiveness for each individual. Primary advantages of TMS remain its potential ease-of-use, relatively low expense, and low risk. Yet the larger question is to what extent NIBS can emerge as a curative therapy on par with medication and destructive approaches. Perhaps an even more salient question is to what extent can lessons learned from rTMS trials in other diseases be applied to epilepsy. From our perspective, the answers remain unknown, but near-future work will likely unmask links between basic mechanisms of neural plasticity and aspects of epilepsy pathophysiology that can be assessed and modulated by TMS and related NIBS techniques.

## Author contributions

Both authors contributed to the conception of the project, writing the initial draft, revising the manuscript, and approving the final manuscript.

## Declaration of generative AI and AI-assisted technologies in the manuscript preparation process

Claude Opus 4.7–4.8 was used to generate initial concepts for Fig. 1 and proofread the manuscript.

## Disclosures and funding

BNL has no personal financial interests. BNL declares intellectual property licensed to Cadence Neuroscience Inc (all contractual rights waived; all funds to Mayo Clinic), site investigator (Rapport Therapeutics, EpiMinder), industry consultant (Epiminder, Livanova, Medtronic, Neuropace; all funds to Mayo Clinic), advisory board member (Livanova, all funds to Mayo Clinic), and data safety board member (Precisis, all funds to Mayo Clinic). BNL is supported by 10.13039/100000002NIH NINDS R01NS129622. AR is co-founder of Galibra, PrevEp and Neuromotion, has received research support from CRE Medical and Neuroelectrics, and is co-inventor on a US patent that pertains to TMS use in epilepsy.

## Declaration of competing and interest

BNL has no personal financial interests. BNL declares intellectual property licensed to Cadence Neuroscience Inc (all contractual rights waived; all funds to Mayo Clinic), site investigator (Rapport Therapeutics, EpiMinder), industry consultant (Epiminder, Livanova, Medtronic, Neuropace; all funds to Mayo Clinic) and advisory board member (Livanova, all funds to Mayo Clinic). BNL is supported by 10.13039/100000002NIH NINDS R01NS129622. AR is co-founder of Galibra, PrevEp and Neuromotion, has received research support from CRE Medical and Neuroelectrics, and is co-inventor on a US patent that pertains to TMS use in epilepsy.
